# Who Are You Calling a Squirt?

**DOI:** 10.1371/journal.pbio.0040243

**Published:** 2006-06-27

**Authors:** Caitlin Sedwick

An enduring mystery that has captivated generations of scientists is the question of humanity's evolutionary origins. It's thought that complicated and specialized creatures such as ourselves evolved from much simpler ancestors by co-opting and elaborating upon genes, proteins, and tissue arrangements pioneered by more-primitive organisms. If that's so, then we should be able to see features reminiscent of ourselves in creatures that look very little like us.

Scientists studying tunicates (sea squirts) have demonstrated several ways in which these organisms can inform us about our own developmental and evolutionary origins. Tunicates are an ancient life form, with fossils dating back to the Neoproterozoic era (542–1,000 million years ago). These marine organisms start out as embryos, then develop into a mobile tadpole form (complete with a brain-equivalent and a tail with a nerve cord and a stiff notochord that functions like a spine) that swims around looking for a substrate to attach to. Once attached, the tail is reabsorbed and the animal assumes a tube-like shape covered in a strong but flexible “tunic.”

To get a better understanding of the molecular events that underlie the development of the vertebrate peripheral nervous system (PNS)—neural structures outside the brain that are important for sensing the environment—Andrea Pasini, Sébastien Darras, and colleagues undertook a systematic characterization of the early developmental events that produce the tunicate tadpole PNS. To do this, they went all the way back to the fertilized egg to examine the origins of cells that contribute to the physical structures of the tadpole tail. With their tadpole tail and spinal-like structures, tunicates are the distant cousins of vertebrates like ourselves.

Following fertilization and three rounds of cell division, which create an eight-cell embryo, two of these eight cells (designated “b4.2”) will ultimately give rise to all the cells that contribute to the external tissues of the tail, including the animal's epidermis and ciliated caudal epidermal sensory neurons (CESNs) of the PNS. By the time the embryo has divided to the size of 110 cells, each b4.2 cell has given rise to 16 cells (called b-line cells). The researchers first looked to see which of these b-line cells contributed to what parts of the tail by labeling individual cells with a fluorescent dye at the 110-cell stage and then looking at the embryos again once tail development was complete. This revealed a remarkably regular organization of the tail epidermis, subdivided into midline, mediolateral and lateral territories, each originating from mostly distinct precursors. In particular, they find that cells that contribute to the dorsal and ventral midline tissues give rise to both epidermis and CESNs.

Pasini et al. found that two secreted factors, fibroblast growth factor (FGF) and bone morphogenetic protein (BMP), respectively specify the dorsal and ventral midline identity of b4.2-derived cells during the first eight hours of development. Interestingly, both FGF and BMP are also involved in the formation of the midline epidermal territories in vertebrates. Once the midline territories are specified, the embryo needs to select among these cells the minority that will form epidermal neurons. In other animals, one pathway that governs the generation of two different cell fates in neighboring cells that derive from a common progenitor cell is the Notch pathway. Indeed, when Pasini et al. looked in tunicates, they found evidence that the Notch pathway controls the number of CESNs that are produced in dorsal and ventral midline tissues. Taken together, the study provides a molecular framework explaining how the embryo sequentially specifies first a bipotential population of cells in the midline epidermis, and then selects among these cells those that will adopt a neuronal fate.

Pasini et al. say that their observations in tunicates can pave the way to a better understanding of how analogous structures form in higher animals; some of the pathways and signaling molecules at work here appear to play similar roles in the development of the vertebrate neural plate border but also, and more unexpectedly, in the formation of the median fin that allows the animal to swim. Thus, the common ancestor of tunicates and vertebrates may have coupled the formation of the fin and its sensory innervation, a coupling lost in modern vertebrates but which remains in the tunicates.

Several questions remain to be clarified in this work; for example, the authors still don't know whether FGF, BMP, or Notch directly control cell-fate decisions, or whether intermediate signaling pathways may be involved. However, they have plenty of precedents to rely on in tracking down the answers to these questions; some of the first insights in experimental embryology came from Laurent Chabry's work in tunicates in 1888, and it is inspiring to think of how much we still have to learn from this animal.

## 

**Figure:**
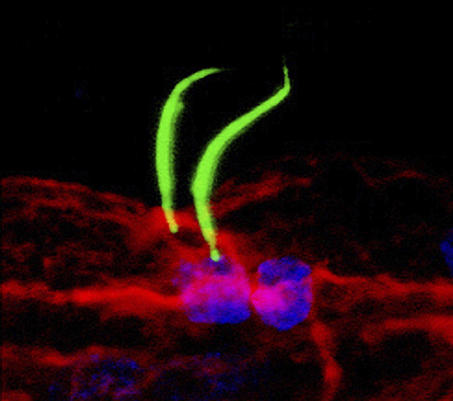
The specification of a pair of sensory neurons present in the *Ciona intestinalis* tail epidermis midlines is a two-step process controlled by the FGF, BMP, and Notch signaling pathways.

